# Strategies for immune regulation in iPS cell-based cardiac regenerative medicine

**DOI:** 10.1186/s41232-020-00145-4

**Published:** 2020-09-29

**Authors:** Kozue Murata, Masaya Ikegawa, Kenji Minatoya, Hidetoshi Masumoto

**Affiliations:** 1 Clinical Translational Research Program, RIKEN Center for Biosystems Dynamics Research, 2-2-3 Minatojima-minamimachi, Chuo-ku, Kobe, Hyogo 650-0047 Japan; 2 Institute for Advancement of Clinical and Translational Science, Kyoto University Hospital, Kyoto, Japan; 3 Department of Life and Medical Systems, Faculty of Life and Medical Sciences, Doshisha University, Kyoto, Japan; 4 Department of Cardiovascular Surgery, Graduate School of Medicine, Kyoto University, Kyoto, Japan

**Keywords:** Induced pluripotent stem cells, Transplantation, Immune regulation, Cardiac regeneration

## Abstract

Cardiac regenerative therapy is expected to be a promising therapeutic option for the treatment of severe cardiovascular diseases. Artificial tissues or organoids made from cardiovascular cell lineages differentiated from human induced pluripotent stem cells (iPSCs) are expected to regenerate the damaged heart. Even though immune rejection rarely occurs when iPSC-derived graft and the recipient have the same HLA type, in some cases, such as tissue transplantation onto hearts, the HLA matching would not be sufficient to fully control immune rejection. The present review introduces recent immunomodulatory strategies in iPSC-based transplantation therapies other than MHC matching including the induction of immune tolerance through iPSC-derived antigen-presenting cells, simultaneous transplantation of syngeneic mesenchymal stem cells, and using the universal donor cells such as gene editing-based HLA modulation in iPSCs to regulate T cell compatibility. In addition, we present future perspectives for proper adjustment of immunosuppression therapy after iPSC-derived tissue/organoid-based cardiac regenerative therapies by identifying biomarkers monitoring immune rejection.

## Introduction

Cardiovascular diseases are leading causes of death and medical expenditure worldwide even recent progresses in medical treatments [1]. Therapeutic modalities in severe heart diseases should be further investigated considering that chances in heart transplantation are quite limited due to donor shortage [2], and ventricular assist devices for circulatory support are not anticipated for long-term use due to complications such as thromboembolism, hemorrhage, and infection so far [3, 4]. Regenerative medicine is an emerging therapeutic option to provide new approaches in current cardiovascular medicine [5–8]. One of the key concepts in cardiac regenerative medicine is the supplementation of exogenous stem cells and the derivatives in cardiovascular cell lineages to restore damaged heart tissue structure and function [7, 9–13]. Pluripotent stem cells are expected to be promising cell sources in regenerative medicine for various intractable diseases by virtue of the theoretically infinite proliferative capacity and ability to differentiate into various types of somatic cells [14]. Especially, human induced pluripotent stem cells (iPSCs) are expected to mitigate immune rejection after cell/tissue transplantation in autologous or human leucocyte antigen (HLA)-controlled allogeneic use which has been investigated in various animal allogeneic transplantation models [15, 16] and clinical studies. Immune [17] regulation in human iPSC-based cardiac regenerative therapy allowing graft survival without immunological complications should be further investigated and clinically validated for the standardization of human iPSC-based regenerative medicine in the future. In the present review, we introduce possible strategies for immune regulation in human iPSC-based cardiac regenerative therapy.

## Possible immunological problems in iPSC-based cardiac regenerative therapy

An immunological privilege of iPSCs in regenerative medicine is that autologous iPSCs which are considered to be immunologically identical with the host can be established from the host somatic cells and used for transplantation therapies [17]. However, the establishment of autologous iPSCs from each patient validated for clinical use would be costly and time-consuming which would hamper the standardization of the therapy. Alternatively, Kyoto University and the collaborators have been promoting a project to prepare and stock clinical-grade allogeneic iPSCs from the lymphocytes of HLA-homozygous healthy donors which would be less susceptible for immune rejection [18]. Established stocks of HLA-homozygous iPSCs were reported to cover approximately 32% of the Japanese population for use in clinical studies for various target diseases by 2018 [18].

Several allogeneic transplant models have been tested to validate whether the use of HLA-homozygous iPSCs can mitigate immune rejection. The experiments using cynomolgus monkeys demonstrated that immune rejection is rarely detected in allograft transplantation differentiated from major histocompatibility complex (MHC) type-matched iPSC. In transplantation experiments of retinal pigment epithelial (RPE) cells derived from monkey MHC-homozygous iPSCs, fair engraftment of RPE cells transplanted to MHC-matched animal models was confirmed without the use of immunosuppressants [15]. Another report showed that the dopamine neurons differentiated from iPSCs derived from cynomolgus macaques with homozygous MHC haplotypes were transplanted to monkeys in which at least one of the alleles was identical to the allografts. Consequently, fair survival of grafted MHC-matching dopamine neurons was observed along with the suppression in the accumulation of microglia and lymphocytes into the grafts [16]. In iPSC-based cardiac regeneration, Shiba et al. reported an allogeneic transplantation model using cynomolgus monkeys (*Macaca fascicularis*). iPSCs from MHC-homozygous animals were differentiated into cardiomyocytes (iPSC-CMs) and subsequently transplanted to MHC-matched monkeys by direct intramyocardial injection. The grafted cardiomyocytes showed electrically coupling to the host heart and survived without immune rejection in monkeys treated with clinically relevant doses of immunosuppressants, whereas the transplantation of iPSC-CMs to MHC-mismatched monkeys even treated with immunosuppressants exhibited immune rejection of grafted cardiomyocytes with severe infiltration of T lymphocytes [19].

On the other hand, the benefits of MHC-matching seem to be alleviated in transplantation of iPSC-derived bioengineered heart tissues which is a promising strategy in cardiac regenerative medicine to promote therapeutic efficiency [20–23]. Kawamura et al. reported a cynomolgus monkey-based allogeneic transplantation experiment using cell sheets prepared from iPSC-CMs. In the experiments, monkeys with immunosuppressants could show fair engraftment of iPSC-CM sheets regardless of MHC-matching, whereas even MHC-matched iPSC-CM sheets could not be sufficiently engrafted without immunosuppressants [24]. The results in allogeneic transplantation would be attributed by the presence of minor antigens and may indicate that the significance of MHC matching would be attenuated in iPSC-based cardiac regenerative therapy. In iPSC-based cardiac regenerative therapy through heart tissue transplantation, it might be assumed that strategies beyond HLA-matching would be required for broad prevalence of the transplantation therapy.

## Emerging strategies for immune regulation in iPSC-based transplantation

**Fig. 1:**
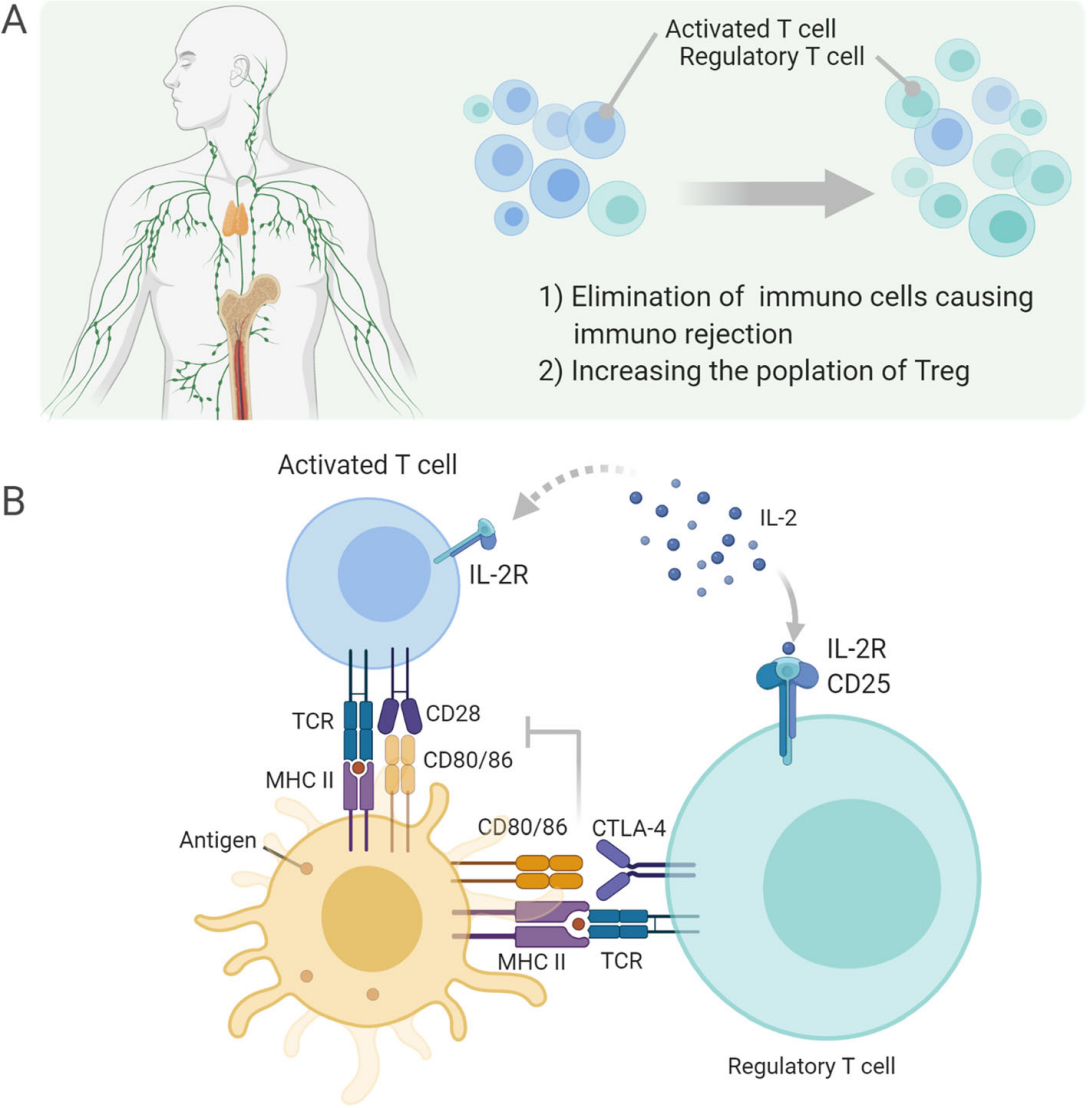
Mechanisms of immunosuppression induced by Treg. **a** Treg establishes immune tolerance by inhibiting the activation and proliferation of immune cells that cause immune rejection. In addition, the high sensitivity of IL-2 causes Treg to selectively proliferate and increases the Treg population. **b** IL-2 is a cytokine essential for T cell differentiation, proliferation, and maintenance and binds to IL-2R; IL-2 is more likely to bind in the presence of CD25; Treg expresses high levels of CD25; Treg reduces the expression of CD80/86 in antigen-presenting cells. In addition, Treg suppresses the CD80/86 co-stimulation to T cells as a result of the high expression of CTLA-4, which is prone to bind CD80/86

**Fig. 2:**
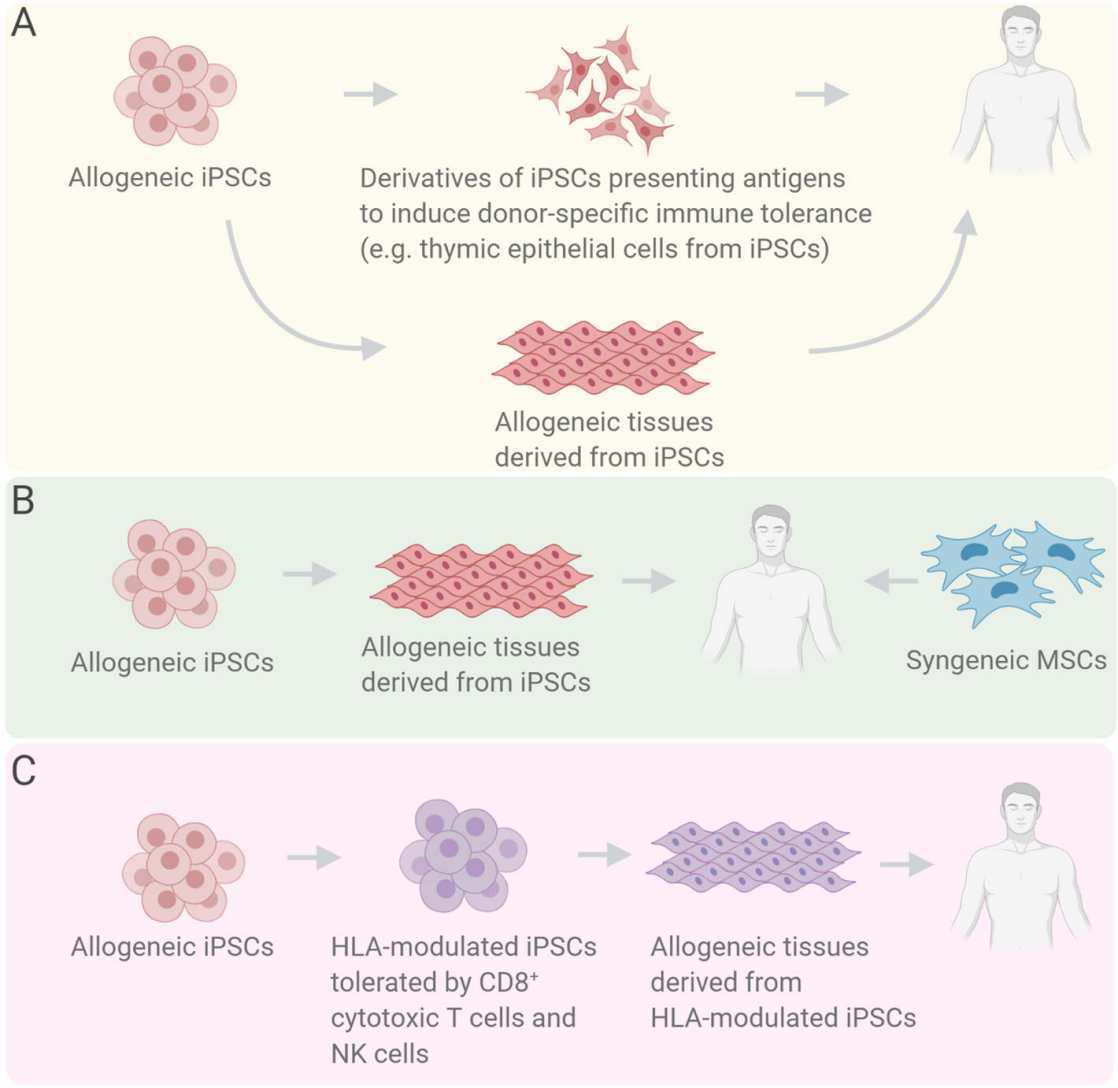
A schema of various immune regulation approaches. **a** Transplantation of thymic antigen-presenting cells prior to iPSC-derived allogeneic cells/tissue to induce donor-specific immune tolerance. **b** Simultaneous transplantation of iPSC-derived allogeneic cells/tissue and syngeneic mesenchymal stem cells (MSCs) to induce immune tolerance. **c** Modulation of HLAs of allogeneic iPSCs to circumvent CD8^+^ cytotoxic T cells and natural killer cells (NK cells)-mediated immune responses

In this context, several attempts have been made to overcome immune rejection through strategies other than HLA-matching so far. One of the promising strategies is the induction of immune tolerance in iPSC-based transplantation. There are two pathways in immune tolerance: central pathway by selective elimination of self-antigenic immune cells in the thymus and bone marrow and peripheral pathway. An important mechanism for this peripheral pathway of immune tolerance is the immunosuppression mediated by regulatory T cells (Tregs) (Fig. 1a). Tregs have T cell receptors and receive antigen presentation, but do not produce IL-2, which is important for T cell differentiation, proliferation, and maintenance. On the other hand, Tregs express CD25, a receptor with high affinity for IL-2. In addition, they express cytotoxic T lymphocyte antigen-4 (CTLA-4) which competitively inhibits the binding between active T cells and CD80/86 of antigen-presenting cells. Based on these features, immune tolerance is established by suppressing T cell co-stimulation and IL-2-induced proliferation of active T cells (Fig. 2b) [25, 26]. The induction of graft-specific Treg-dependent peripheral immune tolerance in liver transplantation is reported to improve the graft viability without the use of immunosuppressants [27, 28]. In iPSC-based transplantation therapies, it would be a possible approach to establish immune tolerance by increasing the total number of Tregs or augmenting the proportion of Tregs through elimination of immune cells causing immune rejection.

Therapeutic approaches for the establishment of immune tolerance are reported so far (Table 1). Otsuka et al. efficiently differentiated thymic epithelial cells from iPSCs (iPSC-TECs) which worked as thymic antigen-presenting cells to induce donor-specific immune tolerance. In an allogeneic transplantation model, transplantation of iPSC-TECs in advance of the transplantation of skin allografts with identical MHCs with iPSC-TECs resulted in prolonged graft survival (Fig. 2a) [29].

**Table 1:** Strategies of immune regulation other than HLA-matching

Author	Strategy	Graft	Recipient	Mechanism of immune tolerance	Ref.
Otsuka et al.	Transplantation with iPSC-derived immunogenicity cells	Tissues derived from iPSCs	Allogeneic animal	iPSC-derived antigens induce donor-specific immune tolerance	[29]
Yoshida et al.	Transplantation with syngeneic MSCs	Tissues derived from iPSCs	Allogeneic animal	Syngeneic MSCs induce Treg cells and the apoptosis of CD8^+^ T cells for graft	[30]
Xu et al.	Universal cells by genomic editing	Tissues derived from HLA-modulated iPSCs	Allogeneic animal	Inducing tolerances from CD8^+^ cytotoxic T cells and NK cells	[31]
Rong et al.	Universal cells by genomic editing	Cells derived from immune-modified ESCs	Allogeneic animal	The expression of immunomodulatory transgenes	[32]

In addition to the induction of immune tolerance by antigen-presenting cells of identical MHC type, tolerance induction using syngeneic mesenchymal stem cells (MSCs) has also been reported (Fig. 2b). Yoshida et al. revealed that simultaneous transplantation of iPSC-derived transplants and syngeneic MSCs increased the expression of anti-inflammatory cytokines TGFβ and IL-2 and then induced Treg. In addition, the simultaneous transplantation of MSCs accelerated the apoptosis of CD8^+^ cytotoxic T cells against the transplants which accordingly achieved the induction of immune torelance [30].

Another intriguing approach is HLA modulation in iPSCs to regulate T cell compatibility which depends on HLA types (Fig. 2c). Xu et al. used a gene-editing technology with CRISPR-Cas9 system to selectively deplete HLA-A and HLA-B on chr.6 of iPSCs to prevent immune responses from CD8^+^ cytotoxic T cells. The modulated iPSCs were also protected from immune responses from natural killer cells because HLA-C, HLA-E, HLA-F, and HLA-G were preserved. Differentiated cells from the modulated iPSCs would be considered as an immunologically ideal cell source for transplantation therapies with enhanced immunocompatibility suppressing immune rejection after transplantation. It is also estimated that only 12 strains of iPSCs with HLA modulation theoretically cover more than 90% of the population worldwide which encourages the establishment of clinical-grade human iPSC bank covering almost all mankind [31]. Even though considerations on off-target mutation risk regarding gene editing would be required, this approach might be a promising strategy towards standardization of iPSC-based transplantation medicine.

CRISPR-Cas9 and other gene-editing systems have recently been used to create donor cells that escape interference from the immune system (universal donor cell). As previously described, it is not only possible to genetically modify the expression patterns of HLA class I and class II, but also to apply the immune escape mechanisms that occur in nature, such as cancer cells and placentas, to prevent the expansion of T cells and NK cells and promote Treg cell responses. Recently, some groups reported the creation of universal donor cells expressing immunomodulatory transgenes that have improved viability in allogeneic transplants [32]. On the other hand, there is a concern in the clinical use of universal donor cells because the universal donor cells might be less sensitive for the elimination through native immune systems in the case of malignant transformation or viral infection. Risk-hedging strategies would be anticipated for the clinical implementation of the universal donor cells such as the transduction of a suicide gene to allow malignantly transformed cells to be eliminated [33].

## Considerations for immune regulation on iPSC-based cardiac regenerative therapy through transplantation of bioengineered heart tissue

Although aforementioned approaches may possibly solve the problems in iPSC-based transplantation immunity, it remains to be further investigated whether these approaches will sufficiently work in iPSC-based cardiac regenerative therapies considering possible severe immunological circumstances of the heart. Another immunological consideration in iPSC-based cardiac regenerative therapies is the possible transition of transplantation subjects from “cells” to “tissue/organoids” [34]. This paradigm shift would be much conceivable in cardiac regenerative therapy because the heart organ works as a pump based on highly organized cell-cell and cell-extracellular matrix assembly, and tissue/organoid structures beyond single cells are required to reproduce heart pump function in future cardiac regenerative therapies.

It also means that immune regulation strategies which would even work in organ transplantation would be required in future iPSC-based cardiac regenerative therapies based on iPSC-derived tissue/organoid transplantation. Given the long history of research on immune rejection in organ transplants [35], it would be predicted that immunosuppressants are somehow required for successful transplantation therapies. It also means that strategies to reduce complications caused by long-term administration of immunosuppressants such as carcinogenesis and infectious diseases [36, 37].

**Fig. 3:**
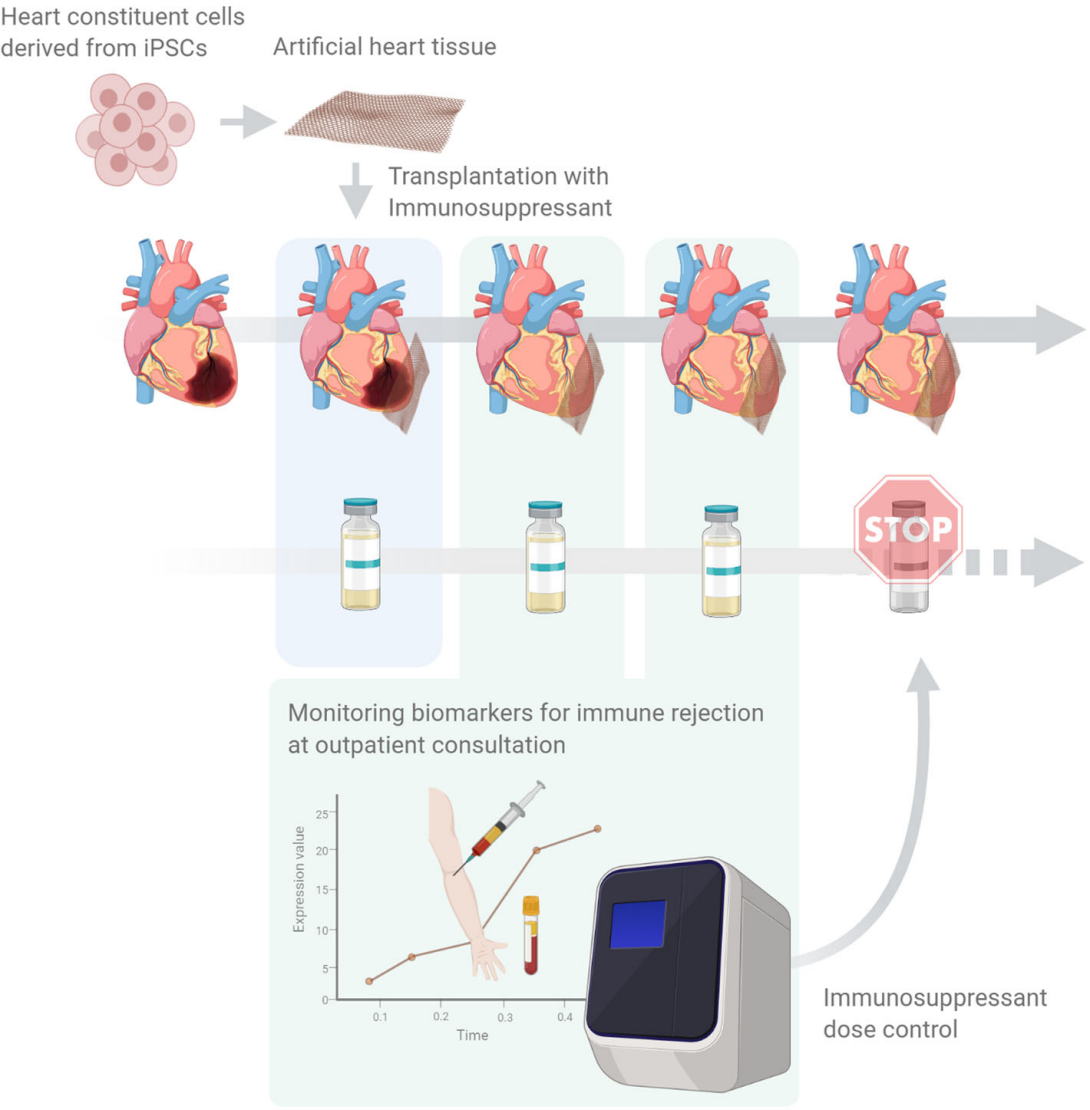
A scheme of the interventional control for immunosuppressive therapy after iPSC-derived heart tissue transplantation. To establish a system to detect immune rejection in daily clinical practices, identification of biomarkers reflecting immune rejection would be anticipated by histological and biochemical evaluations for samples with various immune rejection level

A strategy for the alleviation of immunosuppressant-related complications is to establish a system that can detect immune rejection in daily clinical practices to precisely control the immunosuppression level (Fig. 3). To construct this system, It is necessary to identify specific biomarkers which indicates the occurrence of immune rejection which is not identified so far in the field of iPSC-based transplantation therapy. It is presumed that histological and biochemical evaluations for samples with various immune rejection level controlled by MHC-matching and dose of immunosuppressants obtained through allogeneic transplant model experiments would contribute to identify biomarkers to detect immune rejection dedicated for pluripotent stem cell transplantation therapy. Interventional control of immunosuppression level in clinics using the detection system might provide safer iPSC-based tissue/organoid transplantation therapies for heart diseases.

## Conclusion

In the present review, we introduced present status and possible immunological problems in iPSC-based transplantation therapies including ongoing research attempts for immune regulation such as production of “cells that control the immune system” and “cells that get past the immune system.” Further investigations on immune regulation are required for standardization of iPSC-derived cardiac regenerative therapy based on distinctive features of iPSC-based tissue/organoid transplantation of the heart.

## Data Availability

Not applicable.
